# A bi-stable feedback loop between GDNF, EGR1, and ERα contribute to endocrine resistant breast cancer

**DOI:** 10.1371/journal.pone.0194522

**Published:** 2018-04-03

**Authors:** Sachi Horibata, Edward J. Rice, Hui Zheng, Chinatsu Mukai, Tinyi Chu, Brooke A. Marks, Scott A. Coonrod, Charles G. Danko

**Affiliations:** 1 Baker Institute for Animal Health, College of Veterinary Medicine, Cornell University, Ithaca, NY, United States of America; 2 Department of Molecular Medicine, College of Veterinary Medicine, Cornell University, Ithaca, NY, United States of America; 3 Graduate Field of Computational Biology, Cornell University, Ithaca, NY, United States of America; 4 Department of Biomedical Sciences, College of Veterinary Medicine, Cornell University, Ithaca, NY, United States of America; University of South Alabama Mitchell Cancer Institute, UNITED STATES

## Abstract

Discovering regulatory interactions between genes that specify the behavioral properties of cells remains an important challenge. We used the dynamics of transcriptional changes resolved by PRO-seq to identify a regulatory network responsible for endocrine resistance in breast cancer. We show that GDNF leads to endocrine resistance by switching the active state in a bi-stable feedback loop between GDNF, EGR1, and the master transcription factor ERα. GDNF stimulates MAP kinase, activating the transcription factors SRF and AP-1. SRF initiates an immediate transcriptional response, activating EGR1 and suppressing ERα. Newly translated EGR1 protein activates endogenous GDNF, leading to constitutive GDNF and EGR1 up-regulation, and the sustained down-regulation of ERα. Endocrine resistant MCF-7 cells are constitutively in the GDNF-high/ ERα-low state, suggesting that the state in the bi-stable feedback loop may provide a ‘memory’ of endocrine resistance. Thus, we identified a regulatory network switch that contributes to drug resistance in breast cancer.

## Introduction

Discovering the molecular basis by which cells specialize in diverse morphological or behavioral phenotypes remains a central challenge in biomedical research. It was nearly 80 years ago now that Conrad Waddington first recognized that cells carry a layer of information independent of their genome sequence that governs cellular behavior and morphology [1,2]. This layer of so-called “epigenetic” information has more recently been interpreted as capturing the abundance of mRNA and proteins, as well as the way that these particles interact (reviewed in: [3]). DNA sequence plays a critical role in shaping the interactions between these mRNA and protein factors. Together these sources of information govern how cells interact with their environment, adopt unique morphologies, or carry out their physiological function in the context of an entire organism.

One example in which the specific connections of a regulatory network are of particular biomedical importance is drug resistance in cancer. Endocrine resistance in breast cancer is an excellent example of a drug resistance phenotype. Approximately 75% of breast cancers are positive for estrogen receptor alpha (ERα) at the time of diagnosis. ERα is a transcription factor that controls a mitogenic growth program in breast cancer cells [4,5]. Blocking ERα is an effective therapy for ER+ breast cancer. However, 40–50% of patients develop endocrine-resistance during the course of treatment [6]. Intensive work over the past decade has implicated several growth factor signaling pathways in contributing to endocrine resistance in breast cancer cells [7–12]. For instance, the RET tyrosine kinase signaling pathway correlates with an endocrine resistance phenotype both in patients and in cell models [7–10]. However, we know little about how genes within these signaling pathways interact with one another, and with existing transcriptional programs controlled by ERα to promote endocrine resistance.

Experimental strategies devoted to mapping gene regulatory network interactions remain challenging to apply in practice. To a large extent, this is explained by the highly interconnected nature of gene regulatory networks and the poor temporal resolution of standard genome-wide tools. An emerging strategy for dissecting transcriptional responses to stimuli involves measuring nascent RNA production [13–17]. This approach is sensitive to rapid and dynamical transcriptional changes, allowing target genes to be identified within minutes of activation and hence distinguishing primary and secondary effects [18–22]. Moreover, measuring primary transcription is a general marker that can be used to identify active transcriptional regulatory elements (TREs), including promoters and enhancers, because these elements initiate RNA Pol II transcription [23–28] which are not observed in RNA-seq data owing to rapid degradation by the exosome complex [25,29]. A recent method for detecting nascent transcription by mapping the location and orientation of actively transcribing RNA polymerase, called Precision Run-On and Sequencing (PRO-seq), serves as a powerful assay for both identifying TREs and measuring gene transcription levels [26].

Recognizing these important advantages we used PRO-seq to map the temporal dynamics of changes in RNA polymerase in response to GDNF treatment in MCF-7 breast cancer cells. We collected data at both short (60 min.) and long (24 hours) timescales in order to read-off the gene regulatory network that lies downstream of GDNF-RET signaling. We found that SRF and AP-1 are the transcription factors responsible for transducing the immediate changes in response to GDNF-induced RET activation, and that these factors act downstream of ERK signaling by releasing promoter proximal paused Pol II into productive elongation. Activation of SRF causes breast cancer cells to switch the active state of a bi-stable feedback loop, by transcriptionally repressing ERα and activating the transcription factor EGR1, which in turn leads to the secondary activation of GDNF. Finally, we find that endocrine resistant MCF-7 cells are in a cellular state characterized by high expression of GDNF (GDNF-hi) and low expression of ERα (ER-low). Taken together, our studies demonstrate a novel bi-stable feedback loop that explains how RET-tyrosine kinase signaling leads to resistance to endocrine therapies in breast cancer.

## Materials and methods

### Contact for reagent and resource sharing

Information and requests for reagent and resources (Table 1) can be directed to the Lead Contact Charles G. Danko (dankoc@gmail.com).

**Table 1:** Reagents and resources.

REAGENT or RESOURCE	SOURCE	IDENTIFIER
Antibodies			
anti-p-ERK	Cell Signaling	Cat# 4695
anti-ERα	Santa Cruz	Cat# sc-543
anti-p-ER	Cell Signaling	Cat# 2511
Chemicals, Peptides, and Recombinant Proteins			
(Z)-4-Hydroxytamoxifen (4-OHT)	Sigma-Aldrich	Cat# H7904
Recombinant human GDNF	PeproTech	Cat# 450–10
SUPERase In RNase Inhibitor (20 U/L)	Life Technologies	Cat# AM2694
Protease Inhibitor Cocktail	Roche	Cat# 11836153001
Biotin-11-ATP	PerkinElmer	Cat# NEL544001EA
Biotin-11-GTP	PerkinElmer	Cat# NEL545001EA
Biotin-11-CTP	PerkinElmer	Cat# NEL542001EA
Biotin-11-UTP	PerkinElmer	Cat# NEL543001EA
Sarkosyl	Fisher Scientific	Cat# AC612075000
Trizol	Life Technologies	Cat# 15596–026
Trizol LS	Life Technologies	Cat# 10296–010
GlycoBlue	Ambion	Cat# AM9515
Hydrophilic streptavidin magnetic beads	NEB	Cat# S1421S
RppH	NEB	Cat# M0356S
T4 RNA Ligase 1	NEB	Cat# M0204L
Critical Commercial Assays			
RNeasy Kit	Qiagen	Cat# 74104
High Capacity RNA-to-cDNA	Applied Biosystems	Cat# 4387406
Power SYBR Green PCR Master Mix	Applied Biosystems	Cat# 4367659
Deposited Data			
All genomic data was deposited in GEO and the sequence read archive	Herein	GSE93229
Experimental Models: Cell Lines			
MCF7-B7^TamS^	(Gonzalez-Malerva et al., 2011)	N/A
MCF7-C11^TamS^	(Gonzalez-Malerva et al., 2011)	N/A
MCF7-G11^TamR^	(Gonzalez-Malerva et al., 2011)	N/A
MCF7-H9^TamR^	(Gonzalez-Malerva et al., 2011)	N/A
Sequence-Based Reagents			
Primers for *ACTB*, see STAR Methods	This paper	N/A
Primers for *ESR1*, see STAR Methods	This paper	N/A
Primers for *GDNF*, see STAR Methods	Boulay et al., 2008	N/A
Primers for *EGR1*, see STAR Methods	Fang et al., 2016	N/A
Software and Algorithms			
cutadapt	Martin, 2011	
dREG	Danko et al., 2015	https://github.com/Danko-Lab/dREG
dREG-HD	Manuscript in preparation; This paper	https://github.com/Danko-Lab/dREG.HD;
bigWig software package		https://github.com/andrelmartins/bigWig
Visualization using R	Team, 2010	
BedTools	Quinlan and Hall, 2010	
bedGraphToBigWig program in the Kent Source software package	Kuhn et al., 2013	
DEseq2	Love et al., 2014	
RTFBSDB	Wang et al., 2016	
Cytoscape software package	Shannon et al., 2003	
GraphPad Prism		

The following reagents and resources were used in this study.

### Cell lines and cell culture

Tamoxifen-sensitive (TamS) B7^TamS^ and C11^TamS^ and resistant (TamR) G11^TamR^ and H9^TamR^ MCF-7 cells were generous gift from Dr. Joshua LaBaer [30]. TamS cells were grown in DMEM containing 5% FBS and 1% Penicillin Streptomycin, and TamR cells were grown DMEM containing 5% FBS, 1% Penicillin Streptomycin, and 1 μM (Z)-4-Hydroxytamoxifen (Tamoxifen; Sigma-Aldrich; Cat No. H7904).

### Cell set up and PRO-seq library preparation

TamS and TamR cells were plated in 150 mm dishes in their regular recommended media. After 24 hours, cells were rinsed with PBS three times to remove any residual tamoxifen. Cells were grown in media without tamoxifen for additional three days until approximately 80% confluency. Cells were then treated with 10 ng/mL GDNF for 0, 1, or 24 hours. Cell nuclei were isolated as described previously [17] and nuclear run-on experiments were performed as described previously [13,31] with modifications (see Supplemental Experimental Procedures). PRO-seq library preparation were executed according to Illumina protocol and were sequenced using the Illumina NextSeq500 sequencing.

### Identification of TREs using dREG-HD

TREs were identified using dREG [26]. Data collected between different time points (GDNF treatment) was combined to increase statistical power for the discovery of TREs. We used our dREG-HD [32] to locate precise coordinates of TREs (available at https://github.com/Danko-Lab/dREG.HD).

### Differential expression analysis (DESeq2)

When comparing gene expression in GDNF treated and untreated MCF-7 cells, we counted reads in the window between 1,000 bp downstream of the transcription start site and the end of the annotation or 60,000 bp into the gene body (whichever was shorter). This window was selected to avoid (1) counting reads in the pause peak near the transcription start site, and (2) to focus on the 5’ end of the gene body affected by changes in transcription during 60 minutes of GDNF treatment assuming a median elongation rate of 2 kb/ minute. We limited analyses to gene annotations longer than 2,000 bp in length. To quantify transcription at enhancers, reads on both strands in the window covered by each dREG-HD site were counted. DESeq2 [33] was used for differential gene expression analysis (false discovery rate (FDR) < 0.01).

### Motif enrichment analysis

Motif enrichment analyses were completed using our RTFBSDB as described previously [34]. We used the set of 1,964 human motifs in RTFBSDB clustered into 622 maximally distinct DNA binding specificities, which represents the default settings in the package. The motif that represents each cluster was selected to be the canonical transcription factor that was most highly transcribed in MCF-7 cells. We filtered matches based on a log odds ratio of 7.5 (log *e*) fir a motif match compared with a third-order Markov model background. We identified motifs that were strongly enriched in TREs that change transcription between two conditions compared to a background set. Motifs were evaluated using Fisher’s exact test with a Bonferroni correction for multiple testing. the background set consisted of >1,500 TREs matched for GC content that do not change (<0.25 absolute difference in magnitude (log-2 scale) and *p* > 0.2). We used the enrichmentTest function in RTFBSDB [34].

### Immunoblot analysis

Whole cell lysates were resolved by SDS-PAGE followed by transfer to PVDF membrane. The membranes were stained with Ponceau to visualize the total bound-protein. The membranes were incubated overnight with primary antibodies diluted in TBST in 4°C using the following antibody concentrations: anti-p-ERK (1:1000; Cell Signaling; Cat# 4695), anti-ERα (1:1000; Santa Cruz; Cat# sc-543) and anti-p-ER (1:1000; Cell Signaling; Cat# 2511). The primary antibodies were detected with HRP-conjugated secondary antibodies and were exposed to ECL reagents.

### Pausing analysis

Pause and gene body densities were quantile normalized across all GDNF time course PRO-seq data before pausing analysis in order to avoid potential unknown confounding effects, as described by Danko et. al. (2013). Pausing indices were defined as the ratio of quantile normalized RNA polymerase densities in 500 bp centered on the annotated GENCODE (v19) transcription start sites and the gene body (+1kb to +60kb, as defined above). In the pausing analysis we compared the log *e* transformed ratio of pausing indices between 1 hour of GNDF and untreated TamS MCF-7 cells. All computations were preformed using the R statistical package.

### Reconstructing tamoxifen resistance regulatory network

We defined direct targets of E2 and GDNF signaling as all of those genes undergoing transcriptional changes following short durations of ligand treatment (<40–60 minutes). We used existing GRO-seq data following 40 minutes of E2 treatment (GSE27463). Data following GDNF treatment were collected during the course of this study. Secondary targets were defined as transcriptional changes following 24 hours of GDNF treatment. Networks were visualized using the Cytoscape software package [35].

### Estimating ESR1 start time

First, we estimated the position of the Pol II wave at 60 min. of GDNF treatment to be ~104 kb using a 3 state hidden Markov model [19]. The 60 min in which GDNF was present in the culture media can be represented in two stages: First, we assume a delay, *D*, which is of interest to estimate here, before GDNF signaling induces the repression of *ESR1*. Second, Pol II transcribes for the remaining time, *60 –D*, at an average elongation rate, *r*. The delay *D* can be estimated as:

104000=r×(60−D)

We used two estimates for the elongation rate, *r*, at ESR1: First, we estimated the elongation rate of *ESR1* in MCF-7 cells to be ~1.77 kb/min between 10 and 40 min of E2 treatment [18]; Second, we used an alternative estimate using the median elongation rate in MCF-7 cells of 2.1 kb/min [19].

Solving for the delay *D* at these elongation rates suggests that *ESR1* down-regulation begins between approximately 1.13 min and 10 min, respectively, after adding GDNF to the MCF-7 culture media.

### RNA isolation and quantitative real-time PCR

RNA was purified using the Qiagen RNeasy Kit and reverse-transcribed using the Applied Biosystems High Capacity RNA-to-cDNA kit following the manufacturers’ protocols. Real-time quantitative PCR analysis was performed using the Power SYBR Green PCR Master Mix on *ACTB*, *ESR1*, *GDNF* [36], *EGR1* [37] primers (Table 2). Samples were normalized to β-actin (*ACTB*) and at least three biological replicates were performed. Data are represented as mean ± SEM. Statistical analyses were performed using a two-tailed unpaired Student’s t-test in GraphPad Prism.

**Table 2:** Primer sets.

Target Genes	Primer
*ACTB* Forward	5’-CCAACCGCGAGAAGATGA-3’
*ACTB* Reverse	5’- CCAGAGGCGTACAGGGATAG-3’
*ESR1* Forward	5’- TTACTGACCAACCTGGCAGA-3’
*ESR1* Reverse	5’-ATCATGGAGGGTCAAATCCA-3’
*GDNF* Forward	5’- TCTGGGCTATGAAACCAAGGA-3’
*GDNF* Reverse	5’- GTCTCAGCTGCATCGCAAGA-3’
*EGR1* Forward	5’- AGCCCTACGAGCACCTGAC-3’
*EGR1* Reverse	5’- GTTTGGCTGGGGTAACTGGT-3’

Quantitative real-time PCR was conducted using the indicated primer sets.

### Estimating bi-stable feedback loop state

We derived a score that represents the degree to which cells were dominated by the GDNF or ESR1 state in the bi-stable feedback loop. Intuitively, the score represents the extent to which each cell line recapitulates transcriptional signatures downstream of either GDNF (24 h) or E2 (40 m) by taking the sum of scores across all genes weighted by the magnitude of effect. The score is computed by the following formula:

$$s=\frac{\sum_{g}w_{GDNF}\frac{f-F_{0h}}{F_{24h}-F_{0h}}}{\sum_{g}w_{GDNF}}-\frac{\sum_{g}w_{E2}\frac{f-F_{0m}}{F_{40m}-F_{0m}}}{\sum_{g}w_{E2}}$$

Where *w*_*GDNF*_ and *w*_*E2*_ represent the fold change of each gene, *g*; f represents the RPKM normalized read counts for gene *g* in the sample of interest; *F*_*0h*_ and *F*_*24h*_ represent the mean TamS RPKM normalized read counts after 0 and 24 hours of GDNF treatment; and *F*_*0m*_ and *F*_*40m*_ represent the mean RPKM normalized read counts after 0 and 40 minute treatments with E2. This score is high when the targets of GDNF are activated, and low when the targets of E2 are activated.

### Statistical analysis

Number of biological replicates (n), mean ± SEM, and statistical significance are reported in the Figure legends. Using two-tailed Student’s t-test, data with p < 0.05 are reported statistically significant. In the figures, asterisks (*) and pound (#) signs denote statistical significance. Specific p-values are indicated in the Figure legends. Statistical analyses were performed using GraphPad Prism 7.

### Data and software availability

Raw data files for PRO-seq were deposited to the Gene Expression Omnibus (GEO) under accession number GSE93229. All the software and scripts used in the manuscripts are publicly available on GitHub at https://github.com/Danko-Lab/mcf7tamres; recent version number: 855156ad07c042c88089cb4f31bf9d544487a1b2.

## Results

### GDNF-RET signaling indices a broad transcriptional response to stimuli

We took advantage of an existing MCF-7 model [12,30] to map the transcriptional regulatory interactions downstream of RET tyrosine kinase signaling. We have recently reported that GDNF, a ligand activating RET tyrosine kinase, is necessary for resistance to two endocrine therapies, tamoxifen and fulvestrant, in two MCF-7 subclones (TamR; G11^TamR^ and H9^TamR^) [12]. We have also shown that two clones were highly sensitive to endocrine therapies (TamS; B7^TamS^ and C11^TamS^), which represent the ground-state of ER+ breast cancer cells. Resistance to endocrine therapies can be introduced in B7^TamS^ lines by treatment with recombinant GDNF through activation of the endogenous RET signaling pathway [12].

**Fig 1:**
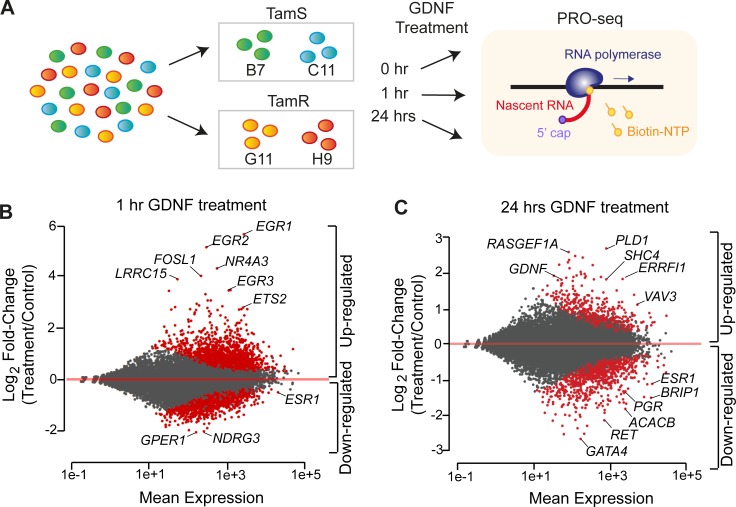
GDNF activates thousands of target genes. (A) Schematic illustration of experimental setup. PRO-seq libraries were prepared from TamS and TamR MCF-7 clones grown in the presence of GDNF for 0, 1, or 24 hours. (B-C) MA plot shows significantly upregulated and downregulated genes (red) following 1 hour (B) or 24 hours (C) of GDNF treatment in TamS MCF-7 cells.

We hypothesized that we could use recombinant GDNF to precisely control the timing of changes in gene expression that ultimately result in endocrine resistance, providing new insights into the responsible pathways. We used PRO-seq to map the location and orientation of RNA polymerase in both TamS and TamR MCF-7 cell lines following induction of RET signaling using recombinant GDNF (10 ng/ ml). To identify both direct and indirect targets, we collected PRO-seq data following a time-course of 0, 1, and 24 hours of GDNF in B7^TamS^, C11^TamS^, G11^TamR^, and H9^TamR^ MCF-7 cells (Fig 1A). We sequenced PRO-seq libraries to a combined depth of 269 million uniquely mapped reads (S1 Table), and confirmed that endocrine sensitive and resistant subclones (B7^TamS^ and C11^TamS^; G11^TamR^ and H9^TamR^) were highly correlated across the time course, supporting the use of separate clones as biological replicates (Spearman’s rank correlation *ρ* > 0.95; S1A and S1B Fig).

Using DESeq2, we found that GDNF treatment changed the transcription of 4,921 annotated (GENCODE v19) transcription units, covering ~15% of expressed transcripts (FDR < 0.01, DESeq2 [33]; Fig 1B and 1C) at either the 1 or 24 hour time points in TamS MCF-7 cells. Most targets were regulated immediately in a burst of transcription following 1 hour of GDNF treatment (n = 3,849 at 1hr). Many genes were rapidly and dramatically activated by 1 hour of GDNF, including immediate early transcription factors *EGR1* and *ETS2* (Fig 1B). Transcription of *ESR1*, the gene that encodes the master transcription factor ERα, was down-regulated (~2-fold) following 1 hour of GDNF. Changes in the transcription of genes induced by GDNF were highly correlated between TamS and TamR cell lines (Pearson’s R > 0.73, *p* < 2.2e-16; S1C and S1D Fig). Transcriptional responses were lower in magnitude in TamR MCF-7 cells following both 1 and 24 hours of GDNF treatment, likely reflecting a dampened GDNF response in TamR lines due to higher basal levels of GDNF acting to stimulate the RET receptor in an autocrine fashion, as expected based on our previous work [12]. We conclude that GDNF causes rapid and extensive changes in transcription at thousands of genes, many of which are likely to kick off a secondary and indirect wave of transcription that explain changes during longer durations of GDNF treatment.

### SRF and AP-1 control enhancer responses to GDNF treatment

**Fig 2:**
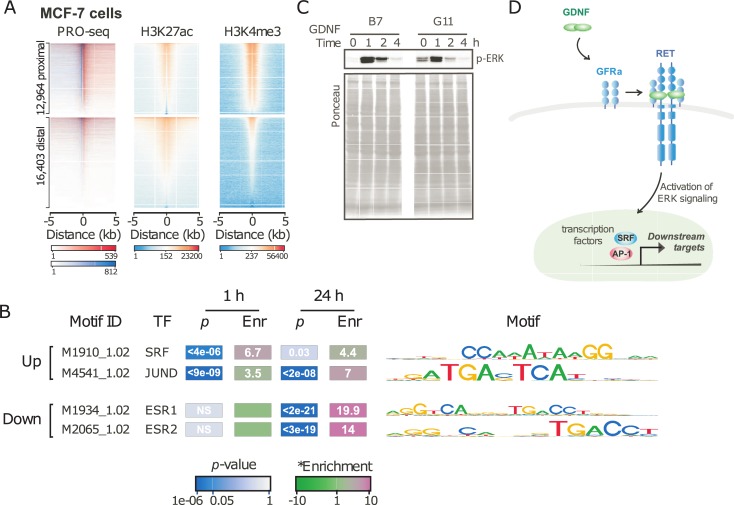
GDNF activates transcriptional regulatory elements. (A) Heatmap depicting PRO-seq, H3K27ac, and H3K4me3 near 1,520 TREs uing dREG from PRO-seq data. (B) Motifs enriched in 1,036 TREs that changed following 1 hr of GDNF treatment compared with TREs that have consistent levels. (C) Immunoblot analysis of p-ERK in B7^TamS^ and G11^TamR^ cells treatment with 10 ng/mL GDNF. (D) Schematic illustration of signaling pathways of tamoxifen resistant MCF-7 cells.

We used dREG [26] to identify the location of 39,119 transcriptional regulatory elements (TREs) that were active during at least one of the GDNF treatment time points. Comparing the location of TREs with histone modifications in resting MCF-7 cells revealed patterns that were characteristic of both promoters and distal enhancers. Whereas both gene distal (>5000 bp) and proximal (<100 bp) TREs were enriched for acetylation of histone 3 lysine 27 (H3K27ac), a mark of both distal enhancers and promoters, gene-proximal TREs were enriched for histone 3 lysine 4 trimethylation (H3K4me3) to a larger extent than distal TREs (Fig 2A). Taken together, these enrichments validate the use of nascent transcription in discovering the location of TREs involved in mediating the GDNF response.

To define the dynamic changes in TRE activities across the time course, we counted PRO-seq reads in a window extending TREs by 500 bp in both orientations to capture paused and elongating RNA polymerase adjacent to the TRE center, and analyzed counts using DESeq2. Our analysis discovered 1,520 TREs with highly confident changes in Pol II loading across the time course (DESeq2, FDR adjusted *p* < 0.01). Discriminative motif discovery using RTFBSDB [34] identified two motifs that were highly enriched in 1,036 TREs that changed following 1 hour of GDNF treatment compared with those transcribed at consistent levels throughout the time-course (Fig 2B). We observed the largest enrichment (8.7-fold) in motifs recognized by serum response factor (SRF) (*p* < 2e-5, Fisher’s Exact Test). In addition to SRF, a motif recognized by AP-1, a heterodimer comprised of FOS, JUN, and ATF family members was also enriched 2.9-fold (*p* < 1e-5, Fisher’s Exact Test).

In neurons, where GDNF is best studied, GDNF activates SRF through the MAPK-ERK signaling pathway [38]. Using Western blotting, we found that ERK phosphorylation is rapidly (2 min.) and dramatically increased in B7^TamS^ MCF-7 cells (Fig 2C). Taken together, these findings support a model in which GDNF exerts its immediate transcriptional effects by the activation of p-ERK and downstream effects on the SRF and AP-1 transcription factor complexes (Fig 2D).

### GDNF releases paused Pol II into productive elongation

**Fig 3:**
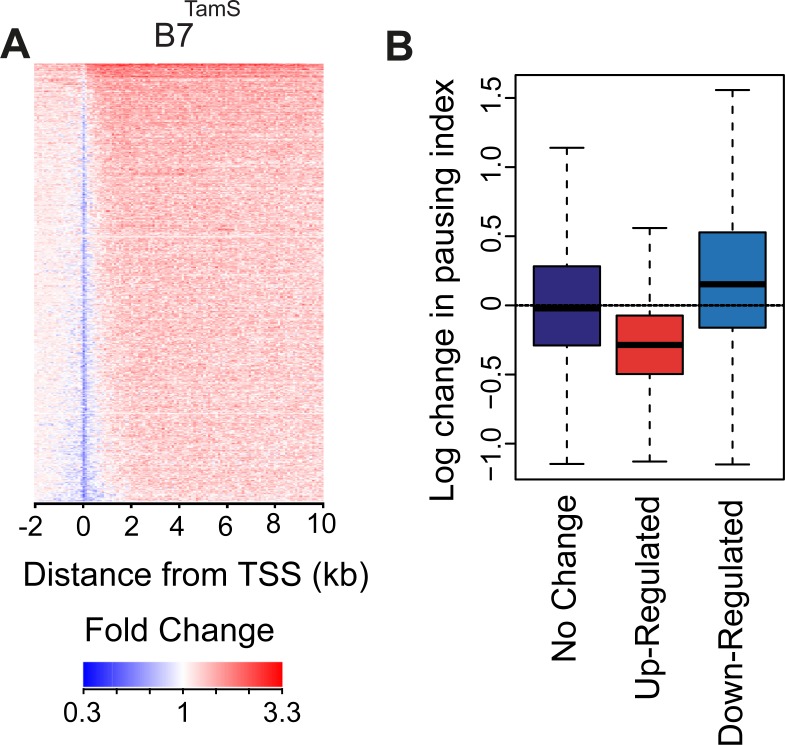
GDNF stimulates the rate at which paused Pol II transitions into productive elongation. (A) Heatmap depicting changes in RNA polymerase density following 1 hour of GDNF treatment in B7^TamS^ MCF-7 cells. (B) Changes in pausing index between treated (1 hour) and untreated TamS MCF-7 cells at the indicated class of genes. The Y-axis represents log base e of changes in read density in the promoter compared to the gene body.

Transcription factors regulate transcription by changing the rates of several steps early during gene transcription (reviewed by [39]). Although Pol II densities increase in the bodies of genes activated by GDNF, the pause peak decreased in both TamS cell lines (Fig 3A), suggesting that GDNF increases transcription, in part, by stimulating the rate at which paused RNA Pol II transitions into productive elongation. To test this hypothesis more rigorously, we computed changes in the pausing index between GDNF-treated (1 hr) and untreated TamS MCF-7 cells at genes up- or down-regulated by GDNF. To avoid potentially confounding batch effects we assumed that global pausing levels were the same in all samples, as described previously [19]. Whereas genes that do not undergo changes in gene body transcription had consistent pausing indices under various conditions, up-regulated genes were observed to have a lower pausing index after 1 hr of GDNF treatment (Fig 3B; *p* < 2.2e-16 Wilcoxon rank sum test). Likewise, down-regulated genes were observed to have slightly but significantly higher pausing indices (*p* < 2.2e-16; Wilcoxon rank sum test). These results suggest that GDNF treatment activates or represses genes in part by changing the rate at which Pol II transitions from a paused state to productive elongation.

### ESR1 and GDNF-EGR1 form a bi-stable feedback loop

Having dissected the factors contributing to the early GDNF response in MCF-7 cells, we set out to define the transcriptional regulatory network downstream of the initial changes in GDNF-RET signaling. Our approach leverages information in the dynamics with which transcriptional changes arise to separate direct and indirect target genes. We compared responses following GDNF to those in response to 17β-estradiol (E2), which activates ERα [18]. We assume that genes up-regulated during the first 40 min. (E2) or 1 hour (GDNF) of treatment are primarily comprised of direct targets because not enough time has elapsed for transcription, translation, and successive rounds of transcriptional activation. Secondary targets responding downstream of GDNF were defined as transcriptional changes following 24 hrs of treatment.

**Fig 4:**
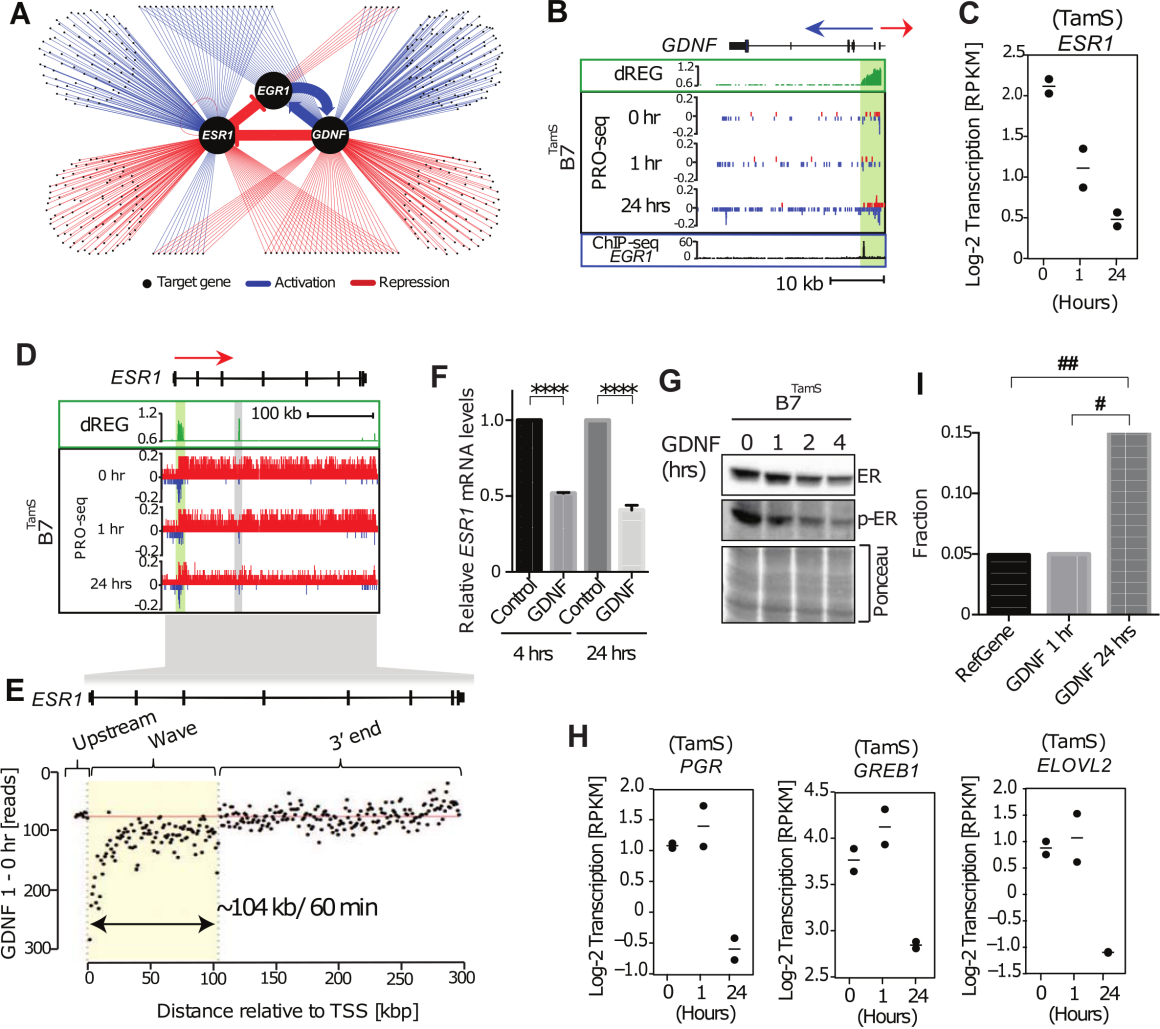
Bi-stable feedback loop between *ESR1*, *EGR1*, and *GDNF*. (A) Transcriptional regulatory network of GDNF-dependent endocrine resistance highlighting the bi-stable feedback loop inferred between *ESR1*, *EGR1*, and *GDNF*. Each point represents a gene regulated following 1 or 24 hours of GDNF signaling. Only transcription factors or signaling molecules are shown. Blue and ref edges represent activation or repression relationships, respectively. (B) Transcription near the *GDNF* locus in B7^TamS^ cells. PRO-seq densities on sense strand and anti-sense strand are shown in red and blue, respectively. dREG scores are shown in green. The promoter is shown in light green shading. Arrows indicate the direction encoding annotated genes. (C) Dot plots of transcription levels of *ESR1* following GDNF treatment. (D) Transcription in the *ESR1* gene in B7^TamS^ cells. PRO-seq densities on sense strand and anti-sense strand are shown in red and blue, respectively. dREG scores are shown in green. Enhancers and promoters are shown in grey and light green shading, respectively. Arrow indicates the direction encoding annotated genes. (E) Difference in read counts in 3kb windows along *ESR1* between 1 hours of GDNF and untreated TamS MCF-7 cells. The location of the wave of RNA polymerase along *ESR1* was identified using a hidden Markov model and is represented by the yellow box. (F) *ESR1* mRNA expression levels in B7^TamS^ cells following 10 ng/mL GDNF treatment. Data are represented as mean ± SEM (n = 3). **** p <0.0001. (G) Immunoblot analysis of ERα and p-ERα in B7^TamS^ cells treatment with 10 ng/mL for 0, 1, 2, and 4 hours. (H) Dot plots representing transcription levels of *ERα* target genes (*PGR*, *GREB1*, and *ELOVL2*) following a time course of GDNF treatment. (I) Bar plot showing the fraction of genes whose transcription is up-regulated by 40 min. of E2 in all RefSeq annotated genes (left) or those which are downregulated by 1 (center) or 24 hours (right) of GDNF treatment. E2 target genes were enriched in those down-regulated following 24 hrs of GDNF treatment. The Y axis denotes the fraction of genes that are direct up-regulated E2 targets (defined based on Hah et. al. (2011) and also up-regulated in B7^TamS^). # p = 1.098e-10, ## p = 6.556999e-19. Fisher’s exact test was used for statistical analysis.

Propagating these simple rules revealed a transcriptional regulatory network with extensive crosstalk between E2 and GDNF signaling pathways (Fig 4A). The central feature of this network is a bi-stable feedback loop between GDNF/RET and E2/ERα. In this loop, GDNF directly inactivates the transcription of ERα and activates transcription of *EGR1*, which, in turn, activates *GDNF* transcription at 24 hrs (Fig 4A–4D). Conversely, ERα directly inactivates EGR1, which in turn leads to a secondary down-regulation of *GDNF*. Thus, *GDNF* and ERα are indirect target of each other, and activation of either signaling pathway by environmental stimulation reinforces its own activity through a positive feedback loop, both dependent on opposite effects on the transcription factor EGR1. Importantly, these intermediate interactions that comprise the regulatory network were overlooked in previous studies [8,40], owing to their limited temporal resolution (>3 hours) after both direct and indirect target genes had begun to accumulate.

### Validation and refinement of the bi-stable feedback loop

We asked whether the decrease in ESR1 leads to decreased ERα protein abundance, and ultimately lower transcription of ERα target genes. Using the position of the retreating wave of RNA polymerase after 60 min. of GDNF [19], we estimated that down-regulation of *ESR1* begins between 1.13 min and 10 min after adding GDNF to the MCF-7 culture media, confirming that it is a direct target of GDNF signaling in MCF-7 cells (Fig 4E). Analysis of ESR1 transcript and protein abundance revealed that these transcriptional changes propagate into a 2-fold decrease in *ESR1* mRNA abundance and ERα protein level following 2–4 hours of GDNF treatment (Fig 4F and 4G). Finally, analysis of PRO-seq data after 24 hrs of GDNF treatment revealed that the down-regulation of ERα protein results in the transcriptional down-regulation of E2 target genes. Classical targets such as *PGR*, *GREB1*, and *ELOVL2* are not different at 1 hr of GDNF treatment, but transcriptionally down-regulated between two and four-fold following 24 hrs of GDNF (Fig 4H). We confirmed by qPCR that the GDNF-induced decrease in *PGR* mRNA occurs at 24 hrs but not at 4 hrs (S2A Fig). Genome-wide, ESR1 target genes were more than three-fold enriched in the set of genes responding to GDNF at 24 hrs, but not at 1 hr (Fig 4I). Moreover, transcriptional changes at 24 hours of GDNF negatively correlate with 40 min of E2 treatment (Pearson’s R = -0.14; *p* = 4.2e-3). Finally, the ERα binding motif was enriched in TREs that change Pol II abundance following 24 hrs of GDNF treatment (*p* < 1e-9, Fisher’s exact test; Fig 2B). Taken together, these results provide independent confirmation that GDNF-RET signaling down-regulates the E2 regulatory program by decreasing the transcriptional activity of *ESR1* during the first 10 min of GDNF treatment.

**Fig 5:**
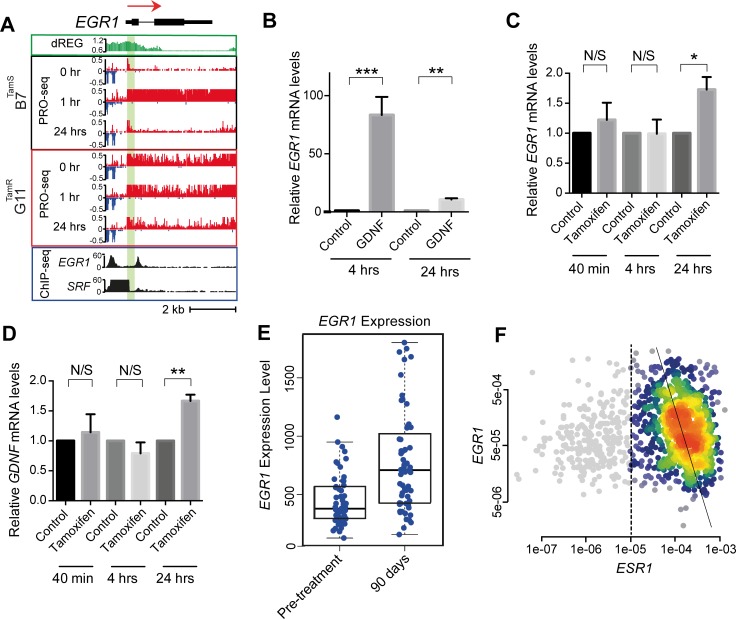
Validation of bi-stable feedback loop in MCF-7 cells and primary breast tumors. (A) Transcription at the *EGR1* locus in B7^TamS^ and G11^TamR^ cells before and after treatment with GDNF. PRO-seq densities on sense strand and anti-sense strand are shown in red and blue, respectively. dREG scores are shown in green. The number of reads mapping in EGR1 and SRF ChIP-seq data is shown in black. Arrow indicates the direction of annotated genes. (B) *EGR1* mRNA expression level in B7^TamS^ cell after treatment with 10 ng/mL GDNF for 4 or 24 hrs. Data are represented as mean ± SEM (n = 3). ** p < 0.01, *** p ≤ 0.001. (C) *EGR1* mRNA expression level in G11^TamR^ cells after treatment without (water) or with 10 ng/mL GDNF for 4 or 24 hrs. Data are represented as mean ± SEM (n = 3). * p < 0.05. (D) *GDNF* mRNA expression levels in G11^TamR^ cells after treatment without (water) or with 10 ng/mL GDNF for 4 or 24 hrs. Data are represented as mean ± SEM (n = 3). ** p < 0.005. (E) Boxplots show *EGR1* expression level before or following 90 days of treatment with letrozole (*p* = 1.8e-6, Wilcoxon Rank Sum Test). (F) Density scatterplots show the expression of *EGR1* versus *ESR1* based on mRNA-seq data from 1,177 primary breast cancers. ER+ breast cancers (n = 925), defined based on ESR1 expression (>1e-5), are highlighted in color. The trendline was calculated using Deming regression in the ER+ breast cancers (Pearson’s R = -0.21; *p* = 2.7e-10).

Next we investigated the positive feedback loop between GDNF and EGR1. We integrated our analysis of PRO-seq data with ChIP-seq in MCF-7 cells from the ENCODE project in order to provide insight into which transcription factors underlie each transcriptional response. First, we confirmed that the 30-fold up-regulation of *EGR1* transcription at 60 min. of GDNF (Fig 5A) led to an 83-fold increase in EGR1 transcript abundance following 4 hrs of GDNF treatment (Fig 5B; *p* < 0.01). We attributed *EGR1* transcriptional activation to an SRF binding site in the *EGR1* promoter using MCF-7 ChIP-seq data (Fig 5A), consistent with motif discovery analyses implicating SRF in the early activation downstream of GDNF. Analysis of EGR1 ChIP-seq revealed a binding site in the *GDNF* promoter (Fig 4B), suggesting that secondary activation of *GDNF* works through initial activation of EGR1. This data suggests that SRF activated by ERK signaling directly up-regulates *EGR1* in MCF-7 cells, leading to a positive feedback loop with GDNF.

Our proposed bi-stable feedback loop model predicts that inhibition of ERα should result in increases in both *EGR1* and *GDNF* at the transcriptional level. We confirmed that blocking ERα using tamoxifen significantly increased both *EGR1* and *GDNF* mRNA levels following 24 hours in B7^TamS^ MCF-7 cells (Fig 5C and 5D). Moreover, analysis of public data profiling gene expression in breast cancer patients after blocking ERα using the aromatase inhibitor letrozole [41] found an increase in EGR1 transcript abundance (*p* = 1.775e-06, Wilcoxon rank sum test; Fig 5F), suggesting that the interaction between ERα and EGR1 is active in breast cancer tissue, though we observed no change in GDNF. Finally, *EGR1* and *ESR1* mRNA abundance were strongly and negatively correlated in ER+ breast cancers analyzed using TCGA (Pearson’s R = -0.21; *p* = 2.7e-10; Fig 5E).

These results provide independent confirmation of the positive feedback loop we proposed based on an analysis of PRO-seq data. Taken together, our results demonstrate that GDNF-RET and ERα form a bi-stable feedback loop dependent on EGR1, in which either ERα or GDNF/RET signaling can remain at a high level.

### TamR MCF-7 cells are constitutively in the GDNF-hi ERα-low state

**Fig 6:**
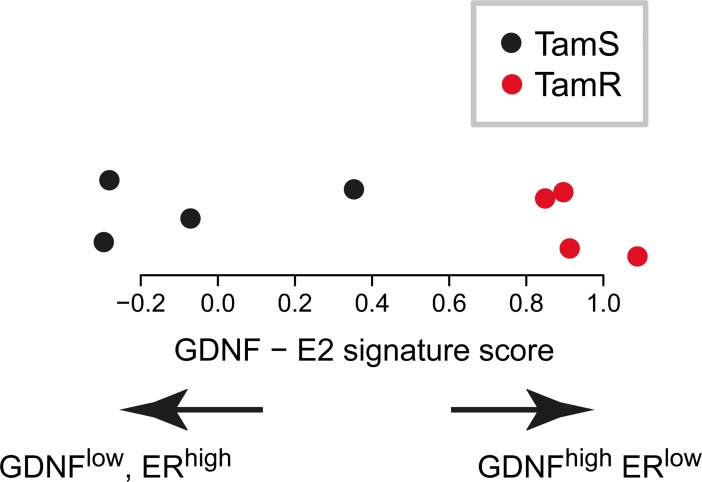
Tamoxifen resistant MCF-7 cells are biased for signatures associated with GDNF activation. (A) Scores summarizing the expression similarities between TamS (black) and TamR (red) cells to gene expression targets that lie downstream of either ERα or GDNF.

We have previously reported that GDNF is sufficient to induce endocrine resistance in TamS MCF-7 cells, and is necessary to maintain resistance in TamR MCF-7 cell lines [12]. Having shown that GDNF switches the active state of a bi-stable feedback loop between GDNF and ERα, we hypothesized that TamR cells are constitutively in the GDNF-hi, ERα-low state. To test this hypothesis, we analyzed PRO-seq data from two separate replicates of all four TamR and TamS cell lines grown in identical conditions for 36 hours. GDNF was transcribed 23-fold higher in TamR than in TamS lines (FDR corrected *p* = 1e-5, DESeq2) [12], suggesting that TamR MCF-7 cells share more similarity with the GDNF-high side of the bi-stable feedback loop. To provide genome-wide support, we devised a score that summarizes the expression similarity of each MCF-7 clonal line to gene expression targets that lie downstream of either GDNF or ERα (see Materials and methods). These scores revealed that TamS MCF-7 cells have a signature that is similar to ERα activation, whereas TamR lines are biased for signatures associated with GDNF activation (Fig 6A). Finally, we also observed constitutive phosphorylation of ERK in G11 TamR lines that was not observed in TamS (Fig 2C) despite consistent transcription levels of both genes in these lines (S3 Fig). This result suggests that endogenous GDNF keeps the MAPK/ ERK signaling pathway constitutively active by signaling RET in an autocrine fashion. Taken together, these results imply that TamR lines exhibit gene expression and cell signaling properties associated with the GDNF-EGR1 arm of the bi-stable feedback loop, whereas TamS cells are driven by ERα.

## Discussion

We have used genomic tools to reconstruct a regulatory network that contributes to endocrine resistance in an MCF-7 breast cancer model. We distinguish primary from secondary target genes by using PRO-seq to measure nascent transcription over short (≤1 hr) and long (24 hrs) treatments with E2 and GDNF, two stimuli that are central to our proposed resistance network. Our work revealed at least two distinct phases of GDNF pathway activation: An initial response, which is governed by MAP kinase signaling activating the transcription factors SRF and AP-1, and a second indirect response governed by the transcription factors EGR1 and AP-1. Transcriptional changes following GDNF stimulation switch the active state in a bi-stable feedback loop by down-regulating ERα and constitutively expressing GDNF. Taken together with our recent work manipulating GDNF expression in MCF-7 cell lines [12], all data strongly support a causal role of this regulatory network in endocrine resistance. Overall, our study provides mechanistic insights into how growth factor ‘escape pathways’ become activated and change the behavior of ER+ breast cancers in ways that facilitate ERα-independent growth.

The positive feedback loop between GDNF and EGR1 provides mechanistic insights into how the GDNF-RET signaling pathway, long implicated in endocrine resistance [8,9], leads to stable ER-independent growth. We propose that GDNF is transcriptionally activated by EGR1, translated, secreted, and acts in an autocrine fashion to further simulate activation of the RET receptor. In support of this model, endocrine resistant MCF-7 cells constitutively express GDNF and its downstream targets (Fig 6A). Moreover, endocrine resistant cells also have a high intrinsic phosphorylation of ERK, compared with endocrine sensitive cells (Figs 6B and 2C), indicating that the RET receptor is constitutively active in TamR cell lines. Finally, the importance of intrinsic GDNF is also supported by GDNF knockdown experiments, in which the formation of the autocrine loop is prevented, and causing TamR MCF-7 cells to become highly sensitive to endocrine treatment [12].

Stimulating endocrine-sensitive MCF-7 cells with GDNF switches the active state in a bi-stable feedback loop by down-regulating ERα and constitutively expressing GDNF. This result was first identified by our PRO-seq time course data, and we have validated this network loop through extensive experimental manipulation (Figs 4 and 5). This type of feedback loop structure might be a sufficient condition to confer resistance to endocrine therapies on its own. Supporting this possibility are our current and previous observations that GDNF is both sufficient to induce resistance in B7^TamS^ MCF-7 cells, and that endogenous GDNF transcription is necessary for resistance in G11^TamR^ cells [12]. Chemotherapy resistance can arise independently of changes to DNA sequence when cells in a population have gene expression profiles that, although rare in the starting population, confer drug resistance to the cells in these states, causing these states to increase substantially following application of the drug by natural selection [42–45]. The bi-stable feedback loop between GDNF and ERα may be the molecular substrate that leads MCF-7 cells to develop endocrine resistance by this epigenetic mechanism.

A bi-stable feedback loop, such as the one that we have observed here, has a number of properties suggesting that it may be part of the molecular substrate that underlies the formation of stable resistance in this MCF-7 model. Perhaps the most important of these properties is that resistance is a stable state in cells within the population. Endocrine resistant MCF-7 cells are associated with the GDNF-hi state for durations of at least 36 hours (Fig 6). This provides an element of cellular memory which can last for multiple cycles of cell division and results in numerous challenges relating to clinical practice. The bi-stable feedback loop may also suggest the use of novel treatment strategies. For instance, we predict that cells could be manipulated back into a GDNF-low/ ER-hi state given higher doses of E2. This will likely promote higher tumor growth rates, but may re-sensitize tumors to endocrine therapies if they are resistant by this mechanism. Alternatively, by applying endocrine therapies in pulses may also prevent the GDNF-hi state of the bi-stable feedback loop described here.

Changes to DNA sequence may also work to reinforce the GDNF-hi state of the bi-stable feedback loop. The extent to which DNA sequence changes contribute to endocrine resistance by this mechanism remains an important question that is not addressed in the present study. We found no evidence for genetic factors that are known to contribute to endocrine resistance in cell models or in patients. For instance, DNA sequence changes to the amino acid sequence encoding *ESR1* can lead to constitutively active ERα, causing resistance to aromatase inhibitors [46]. TamR lines studied here were also highly resistant to fulvestrant, which works by degrading ERα protein, demonstrating that endocrine resistance reported here is ER-independent [12]. Nevertheless, the present study cannot rule out a DNA sequence mutation elsewhere in the genome as a factor that contributes to endocrine resistance in these TamR MCF-7 cell lines.

Our primary goal in the present study was to identify direct and indirect targets of GDNF signaling, which required us to use concentrations of GDNF that produce a robust response. For this reason, we added recombinant GDNF at a concentration of 10 ng/ mL, as recommended by the manufacturer, and used in prior work [8,9]. Therefore, another limitation of the present study is that the dose of recombinant GDNF used is substantially higher than the concentration of GDNF than produced by G11^TamR^ MCF-7 cells [12]. An important question in future studies is how producing physiological quantities of GDNF affects the resistance network that we introduce here.

Taken together, results reported in this study reveal a regulatory network that is responsible for GDNF-RET-mediated endocrine resistance in MCF-7 cells. Longitudinal clinical studies targeting large cohorts will be required to fully validate the clinical relevance of our proposed mechanism of endocrine resistance.

## Supporting information

S1 Table PRO-seq data collection and sequencing depth. PRO-seq was conducted in the indicated cell clone and biological condition. Raw PRO-seq data were sequenced to the uniquely mapped read depth specified and aligned to the human genome (hg19) using established pipelines.
(DOCX)

S1 Fig Highly correlated transcriptional patterns in biological replicates across the time course. (A) Density scatterplot showing global transcriptional levels between TamS (B7 and C11; top) or TamR (G11 and H9; bottom) MCF-7 cell lines at 0, 1, or 24 hours GDNF treatment. (B) Heatmap shows Spearman’s rank correlation of RNA polymerase abundance of TamS and TamR lines between the indicated samples. Sample order is determined by hierarchical clustering. Color scales show 0, 1, or 24 hours of GDNF treatment (above) or TamS or TamR (right) as shown below the heatmap. (C-D) Scatter plots depict transcriptional changes between TamS and TamR MCF-7 cells following (C) 1 hour or (D) 24 hours of GDNF treatment.
(EPS)

S2 Fig GDNF causes decrease in *PGR* mRNA expression and ERα binding sites. (A) *PGR* mRNA expression level in G11^TamR^ cells after treatment without (water) or with 10 ng/mL GDNF for 4 or 24 hrs. Data are represented as mean ± SEM (n = 3). **** p < 0.0001.
(EPS)

S3 Fig No difference in ERK transcription between TamR and TamS cell lines. The dots represent transcription of ERK in TamS (left) and TamR (right) MCF-7 cells. The Y-axis represents a log-2 scale. The difference in means between TamS and TamR is <25% (*p* = 0.42, as estimated by DESeq2).
(EPS)
