# Supplementary material for: A bi-stable feedback loop between GDNF, EGR1, and ERα contribute to endocrine resistant breast cancer
Source: PLoS One. 2018 Apr 3;13(4):e0194522. doi: 10.1371/journal.pone.0194522 (PMC5882141; doi:10.1371/journal.pone.0194522)
Supplement: S1 Table — PRO-seq was conducted in the indicated cell clone and biological condition. Raw PRO-seq data were sequenced to the uniquely mapped read depth specified and aligned to the human genome (hg19) using established pipelines. (DOCX) [file pone.0194522.s001.docx]

| Cell clone | Endocrine status | GDNF [h] | Mapped reads |
| --- | --- | --- | --- |
| B7 | TamS | 0 | 23650727 |
| B7 | TamS | 1 | 19833669 |
| B7 | TamS | 24 | 23766680 |
| C11 | TamS | 0 | 23764089 |
| C11 | TamS | 1 | 20414276 |
| C11 | TamS | 24 | 22651715 |
| G11 | TamR | 0 | 22342301 |
| G11 | TamR | 1 | 22948560 |
| G11 | TamR | 24 | 21691488 |
| H9 | TamR | 0 | 25301129 |
| H9 | TamR | 1 | 18441145 |
| H9 | TamR | 24 | 24319258 |
| Total |  |  | **269125037** |

**Supplementary Table 1. PRO-seq data collection and sequencing depth**. PRO-seq was conducted in the indicated cell clone and biological condition. Raw PRO-seq data were sequenced to the uniquely mapped read depth specified and aligned to the human genome (hg19) using established pipelines.
